# Fiber rich food suppressed airway inflammation, GATA3 + Th2 cells, and FcεRIα+ eosinophils in asthma

**DOI:** 10.3389/fnut.2024.1367864

**Published:** 2024-05-02

**Authors:** Alicia Schenzel, Adriana Geiger, Elvedina Nendel, Zuqin Yang, Susanne Krammer, Anna Leberle, Ann-Kathrin Brunst, Sonja Trump, Susanne Mittler, Manfred Rauh, Carol I. Geppert, Patrick Tausche, Katja Hohenberger, Ralf J. Rieker, Oliver Schieweck, Sebastian Zundler, Susetta Finotto

**Affiliations:** ^1^Department of Molecular Pneumology, Friedrich-Alexander-University (FAU) Erlangen-Nürnberg, Universitätsklinikum Erlangen, Erlangen, Germany; ^2^Children’s Hospital, Department of Allergy and Pneumology, Friedrich-Alexander-University (FAU) Erlangen-Nürnberg, Universitätsklinikum Erlangen, Erlangen, Germany; ^3^Institute of Pathology, Friedrich-Alexander-University (FAU) Erlangen-Nürnberg, Universitätsklinikum Erlangen, Erlangen, Germany; ^4^Deutsches Zentrum für Immuntherapie (DZI), Erlangen, Germany; ^5^Bavarian Cancer Research Center (BZKF), Erlangen, Germany; ^6^Laboratory of Clinic Medicine, Friedrich-Alexander-University (FAU) Erlangen-Nürnberg, Erlangen, Germany; ^7^Department of Internal Medicine 1, Friedrich-Alexander-University (FAU) Erlangen-Nürnberg, Universitätsklinikum Erlangen, Erlangen, Germany; ^8^Comprehensive Cancer Center Erlangen-EMN (CCC ER-EMN), Erlangen, Germany

**Keywords:** fiber rich food, allergic asthma, Th2 cells, GATA3, FcɛRI, antigen presenting cells, memory T cells

## Abstract

**Background:**

Allergic Asthma is a disease presenting various endotypes and no current therapies act curative but alleviate disease symptoms. Dietary interventions are gaining increasing importance in regulating immune responses. Furthermore, short chain fatty acids (SFCA), as the main products of dietary fiber’s fermentation by the gut bacteria, ameliorate the pathogenesis and disease burden of different illnesses including asthma. Nevertheless, the connection and crosstalk between the gut and lung is poorly understood.

**Objective:**

In this work, the role of high fiber diet on the development of allergic asthma at baseline and after exacerbation of disease induced by respiratory viruses was investigated.

**Methods:**

Hereby, SCFA in serum of asthmatic and non-asthmatic pre-school children before and after airway disease symptoms were analyzed. Moreover, the effect of high fiber diet *in vivo* in a murine model of house dust mite extract (HDM) induced allergic asthma and in the end in isolated lung and spleen cells infected *ex vivo* with Rhinovirus was analyzed.

**Results:**

In this study, a decrease of the SCFA 3-Hydroxybutyric acid in serum of asthmatic children after symptomatic episodes at convalescent visit as compared to asthmatic and control children at baseline visit was observed. In experimental asthma, in mice fed with high fiber diet, a reduced lung GATA3 + Th2 type mediated inflammation, mucus production and collagen deposition and expression of Fc epsilon receptor Ia (FcεRIa) in eosinophils was observed. By contrast, the CD8+ memory effector T cells were induced in the lungs of asthmatic mice fed with high fiber diet. Then, total lung cells from these asthmatic mice fed with either standard food or with fiber rich food were infected with RV *ex vivo*. Here, RV1b mRNA was found significantly reduced in the lung cells derived from fiber rich food fed mice as compared to those derived from standard food fed asthmatic mice. Looking for the mechanism, an increase in CD8+ T cells in RV infected spleen cells derived from fiber rich fed asthmatic mice, was observed.

**Conclusion:**

Convalescent preschool asthmatic children after a symptomatic episode have less serum ß-Hydroxybutyric acid as compared to control and asthmatic children at baseline visit. Fiber rich diet associated with anti-inflammatory effects as well as anti-allergic effects by decreasing Type 2 and IgE mediated immune responses and inducing CD8+ memory effector T cells in a murine model of allergic asthma. Finally, *ex vivo* infection with Rhinovirus (RV) of total lung cells from asthmatic mice fed with fiber rich food led to a decreased RV load as compared to mice fed with standard food. Moreover, spleen cells derived from asthmatic mice fed with fiber rich food induced CD8+ T cells after *ex vivo* infection with RV.

**Clinical implications:**

Dietary interventions with increased content in natural fibers like pectins would ameliorate asthma exacerbations. Moreover, respiratory infection in asthma downregulated SCFA in the gut contributing to asthma exacerbations.

## 1 Introduction

Asthma is one of the most common chronic respiratory diseases associated with airway inflammation, mucus hyperproduction, remodeling and hyperresponsiveness (AHR) (1). These changes result in bronchoconstriction, thickening of airway walls, eosinophilic infiltration, and decreased lung function (2). Symptoms vary from wheezing and coughing to chest tightness and constant shortness of breath. With the increase of industrialization, more and more people are diagnosed with asthma every year (1). Approximately 300 million people worldwide are suffering from the symptoms of this disease, and the prevalence is increasing by 50% every decade. Moreover, worldwide 180.000 deaths related to asthma are reported annually (3). Although the prevalence of asthma is steadily rising and new therapies developed, the basic mechanisms of the disease are still largely unknown (4).

Asthma is divided into two sub-phenotypes: intrinsic (non-atopic) and extrinsic (atopic) asthma. Whereas atopic asthma is triggered by a range of allergens such as pollen, mold, and dust mites, non-atopic asthma normally occurs with cold weather, exercising, infections, or stress. Since atopic asthma is associated as a T helper 2 immune response driven disease, it is also described as T2-type asthma (4–7). The underlining mechanism has been attributed at dysregulated antigen presentation by DCs leading to pathologic T-cell differentiation into Th2 cells (8–11). These Th2 cells secrete proinflammatory cytokines like IL-4, IL-5, IL-9, and IL-13 and also the granulocyte-macrophage colony-stimulating factor (GM-CSF) (11) as well as chemokines which leads to the activation and recruitment of macrophages, eosinophils, basophils, and mast cells. Mast cells also secrete IL-4, IL-5, and IL-13, which mediate eosinophilic inflammation, IgE synthesis and thus promote Th2 immune responses (10). Specifically, the release of antigen-specific IgE is induced by class-switch of B cells which express the surface low affinity receptor FceRII. Moreover, IgE binds to high affinity receptor FceRI expressed on mast cells, basophils and APCs which eventually results in mast cell degranulation and the release of further pro-inflammatory mediators (12). Besides that, IL-5 mediates activation of eosinophils and IL-13 induces goblet cell hyperplasia and excessive mucus production in conducting airways which cause the symptoms of the allergic immune response (13). Up to 85% of asthmatic patients worldwide are allergic to house dust mites (HDM) (Dermatophagoides Pteronissinus) (14, 15). The allergenicity of HDMs is composed by several components, including their fecal pellets, dust, and mite-associated bacterial and fungal spores (14). Thus, this asthma model which includes several sensitization steps and HDM challenges either intranasally or intraperitoneally, might be important for a better understanding of the pathogenesis of Type 2 asthma and helpful for developing further therapies (15).

It is already known that the microbiota plays a fundamental role in the regulation and formation of the host immune system. Previous studies demonstrated that altered gut microbiota can affect the lung and the airways (16–18). Dysbiotic gut microbial composition can lead to a disruption of immune homeostasis inside and outside the gut. This is evident in patients with gastro-intestinal conditions such as the chronic irritable bowel syndrome. These patients are also at higher risk for developing respiratory diseases. Nevertheless, the crosstalk between lung and intestinal tract is still poorly understood. Furthermore, various external and internal factors affect the composition of the intestinal microbiome. These enclose diet, genetics and also age of the host, which lead to altered immune response and disruption of lung homeostasis (19). Thereby, dietary intervention is important in modulating the immune system and therefore a promising therapy option (20). Dietary fibers are associated with lung immune response and have beneficial effects on lung function and inflammation. Dietary fibers are carbohydrates, which consist of soluble and insoluble components. The soluble components are further processed anaerobically through microbes in the intestine by fermentation. The most abundant metabolites made from dietary fiber, also called prebiotics, are termed as short chain fatty acids (SCFAs). SCFAs can locally affect the gut or modulate other organs by distribution to peripheral tissues through the blood system. Prebiotics can be ingested through high fiber diets such as fruits or whole grains and are subsequently degraded by microbes in the gut through carbohydrate active enzymes. SCFAs are then produced in pathways such as the propanediol, acrylate, and succinate pathway (21). Pectin as the main component of plant cell walls belongs to the non-starch polysaccharides, which needs to be digested by microbes in the gut and leads to microbiota-derived increased levels of SCFAs in the human body. With that, pectin shows indirect effects by fermentation and increasing levels of SCFAs, but also direct immunomodulatory effects (22). Human blood sample treated with modified pectin showed an activation of T-cells, B-cells, and NK-cells (23). It was also found that pectin dampens the activation of macrophages and delayed-type hypersensitivity reaction (24). Furthermore, modified pectin decreased IgE-Level production, but not IgG or IgM, in human peripheral blood mononuclear cells (PBMCs) *in vitro* (25) and suppressed hypersensitivity reaction in OVA-sensitized mice (26).

The most abundant SCFAs, which result from fermentation, are acetate, propionate, and butyrate (27–30). High levels of SCFAs are consumed by colonocytes, where some SCFAs enter the bloodstream by passive diffusion or active transport via the proton-coupled monocarboxylate transporter isoform 1 (MCT1) or Na^+^-coupled monocarboxylate transporter 1 (SMCT1) and efflux into blood circulation via monocarboxylate transporters MCT3-5 (30). The molecular mechanisms of SCFAs affecting immune cells are binding of SCFAs to G-protein coupled receptors (GPCRs), diffusion channels, and inhibition of histone deacetyl transferases (HDACs). SCFAs connect to and are sensed by GPCRs such as GPR41 (FFAR3), GPR43 (FFAR2), GPR109A, and olfactory receptor 78 (Olfr78) with different sensitivities, which are coupled on signaling pathways (AMP-K, mTOR, STAT3, MAPK, and NF-kB). SCFAs can also be transported via diffusion channels, like the solute carrier family 16 member 1 (Slc) 16a1 and 5a8, directly into cytoplasm where they can interact with various signaling pathways. Via passive diffusion, SCFAs can directly influence HDAC and HAT enzymes and therefore directly regulate gene expression (5). Therefore, SCFAs can mediate anti-inflammatory effects by acting on different targets.

Thus, this study investigated the effect of an increased intake of dietary fiber on asthma pathogenesis. Moreover, in preschool children cohorts Predicta, SCFA in serum after a symptomatic episodes was analyzed. In a murine model of house dust mite induced asthma, the effects of dietary fiber rich food on immune cells, were analyzed. Moreover, lung cells from these asthmatic mice were infected *ex vivo* with Rhinovirus (RV) RV1b, which belongs to the RV-A species (31, 32). A decreased RV load in lung cells derived from fiber rich fed mice and immunological alterations in their *ex vivo* RV infected spleen was found. In summary, these studies would advance current understanding on the effect of dietary natural fibers on the immune system of asthmatics and control subjects at baseline and during asthmatic episodes of asthma exacerbations after respiratory virus infection.

## 2 Materials and methods

### 2.1 Pediatric cohort PreDicta

The European study PreDicta (Post-infectious immune reprogramming and its association with persistence and chronicity of respiratory allergic diseases) assessed two cohorts of healthy and asthmatic preschool children aged 4–6 years old. The study was approved by the ethics committee of the Friedrich-Alexander University (FAU) Erlangen-Nürnberg, Germany (Re-No 4435) and is registered in the German Clinical Trials Register (www.germanctr.de: DRKS00004914). The recruitment of the cohorts, the inclusion and exclusion criteria and the data collection were described previously (33). Characteristics of the children analyzed in this study are reported in Table 1. Starting 2011, 22 healthy control and 24 asthmatic children which fulfilled several inclusion criteria: apart from a written informed consent from the child’s parents or guardians, the disease of asthma had to be diagnosed within the last 2 years and confirmed by a doctor of the Children’s Hospital in Erlangen. The asthmatic disease should be mild to moderate with persistent severity according to the GINA guidelines (2005). Severe or brittle asthma lead to exclusion, as well as other chronic respiratory diseases (cystic fibrosis, bronchopulmonary dysplasia, and immunodeficiency) except allergic rhinitis. Other exclusion criteria were more than six courses of oral steroids during the previous 12 months, other chronic diseases except atopic eczema or chronic medication use. Moreover, children within the control group may not have a history of asthma or wheezing and atopic illness. For this study, only three control and five asthmatic children were analyzed at recruitment (B0). Additionally 11 asthmatic children were analyzed at convalescent visit (see Table 1).

**Table 1:** Clinical data of the children at the baseline (B0) and convalescent (C) visit controls at B0 (Recruitment visit).

Patient ID	Age at B0 (years)	Gender	Allergic rhinitis	Number of upper respiratory infections in the last 12 months
214	5	Male	No	5.5
218	4	Female	No	2
221	3	Male	No	3

Asthmatics at B0 (Recruitment visit)						
Patient ID	Age at B0 (years)	Phenotype (PRACTALL 2008)	Asthma control (GINA 2009)	Allergic rhinitis	Number of upper respiratory infections in the last 12 months	Treatment
207	5	v, a	Partly controlled	No	2	Steroid
210	6	v	Partly controlled	Yes	10	Non-Steroid
216	5	a,v	Uncontrolled	No	5	Steroid
217	6	a,e,v	Uncontrolled	Yes	4	Steroid
228	5	v	Controlled	Yes	2,5	Non-Steroid
Asthmatics at C1						
201	6	v	Controlled	No	1,5	Steroid
202	6	u	Partly controlled	Yes	5	Steroid
210	6	v	Partly controlled	Yes	10	Non-Steroid
212	5	e,v	Controlled	No	12	Steroid
216	5	a,v	Uncontrolled	No	5	Steroid
223	5	v	Controlled	Yes	8	Steroid
224	4	v	Controlled	Yes	12	Steroid
229	4	v	Controlled	Yes	10	Non-Steroid
230*C2	5	v	Controlled	Yes	4	Non-Steroid
238*C2	4	v	Controlled	Yes	6	Steroid
243	5	v	Uncontrolled	Yes	5	Steroid

A, Allergen induced; v, Virus induced; e, Exercise induced; and u, Unresolved.

### 2.2 Timescale

**Figure 1:**
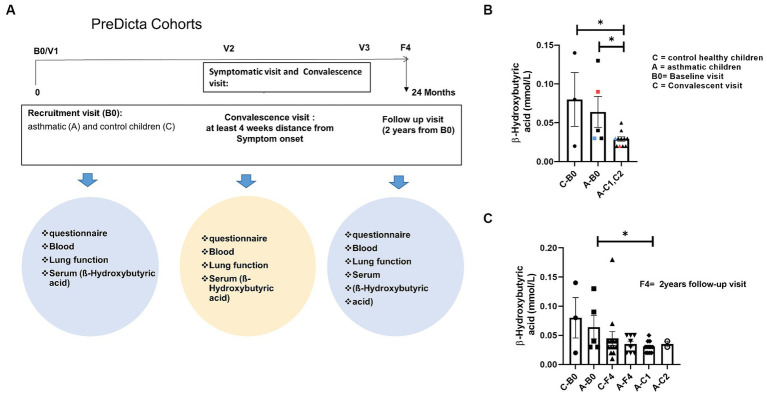
Reduced short chain fatty acid (SCFA) in serum of children with asthma after disease exacerbation. **(A)** Experimental design of the pediatric cohort PreDicta. **(B,C)** Measurement of β-Hydroxybutyric acid in the serum of healthy control children and asthmatic children at the baseline (B0) and convalescent visit (C1, C2) at 6–8 weeks after disease exacerbation (**A**—B0; *n* = 5) vs. **A**—C1, C2 (*n* = 11) *p* = 0.0218; **C**—B0 (*n* = 3) vs. **A**—C1, C2 (*n* = 11) *p* = 0.0109. An unpaired *t*-test was performed to compare the groups; ^*^p < 0.05. All data are shown as mean ± SEM.

Predicta study is a longitudinal prospective evaluation over a 2-year period. At the baseline visit (B0), whole blood was drawn from the children and collected in heparin tubes for serum isolation (Figure 1A). Furthermore, asthmatic children were invited for additional visits within the next 2 days whenever they notice disease symptoms such as respiratory tract infection or cold, an exacerbation of asthma or a decrease of FEV1 > 15% or decreased PEF > 30% (symptomatic visits). Moreover, blood was collected also at the convalescence visit, 6–8 weeks after asthma exacerbations (C1, C2 Visit). In this study, we analyzed serum from selected children because serum was used for previous studies.

### 2.3 Experimental murine model of house dust mite induced allergic asthma

Female 4-week-old BALB/c wild-type mice were purchased from Janvier labs and acclimatized for one and a half weeks before the beginning of the experiment. The mice were housed as four individuals per cage.

Starting on day 0, the mice were fed with two different diet foods: the control groups received standard maintenance diet for mice (1,324, Altromin Spezialfutter GmbH & Co. KG, Lage, Germany), while the test groups received a crude fiber rich diet modified with 30% Pectin (C1014, Altromin International, Lage, Germany) which resulted in a composition of 200.63 mg/kg crude fiber, 105.02 mg/kg Polysaccharides, and 162.59 mg/kg crude proteins. In contrast to this high fiber diet, standard diet contained 6.1% (60.74 mg/kg) Crude fiber, 391.216 mg/kg Polysaccharides, and 192.11 mg/kg crude proteins. The experiments were approved by the local animal ethics committee Regierung von Unterfranken (AZ: 55.2.2-2532-2-633).

For inducing asthma, the mice were sensitized first with 12.5 μg house dust mite extract (HDM) (Stallergenes Greer Laboratories Inc., Lenoir, United States) in 200 μL PBS intraperitoneally on day 12 and 19. The mice were challenged on days 26, 30, and 33 by applying 125 μg HDM intranasally after anesthetizing the mice briefly with isoflurane. The control groups received PBS instead. On day 35, the lung function was measured and the experiment was terminated.

### 2.4 Measurement of 3-Hydroxybutyric acid

The concentration of ß-Hydroxybutyric acid in serum samples was measured by using a kit from Dia Sys, diagnostic systems. Diagnostic reagent for quantitative *in vitro* determination of β-Hydroxybutyrate in serum or plasma on photometric systems (Cat. No. Kit size 1 3711 99 10 930 R1.;21 FS*) uses Enzymatic determination with β-Hydroxybutyrate-dehydrogenase. The absorbance at 340 nm is proportional to the β-Hydroxybutyrate concentration in the sample (Supplementary Table E1).

### 2.5 Flow cytometry staining

After cell isolation from the lungs, spleen, and lymph nodes, the cells were separated and pre-incubated with murine Fc-Block (Cat: 564220, BD Biosciences, Heidelberg, Germany) to prevent unspecific bindings. All staining’s for surface markers were performed at 4°C for 15–20 min in the dark. For phenotyping, mast cells, dendritic cells, Th2 and Th1 cells, regulatory T cells, and memory T cells, the antibodies listed in repository Supplementary Table E2 were used.

For intracellular staining, surface-stained cells were fixed and permeabilized with Fixation/Permeabilization buffer Set (eBioscience, Thermo Fisher Scientific, Waltham, MA, United States) followed by the intracellular staining of the cells at RT for 30 min in the dark. The analysis of the cells was performed on a Flow Cytometer FACS Canto II (BD Biosciences, Heidelberg, Germany). The flow cytometry data were analyzed using FlowJo v10 software (TreeStar Inc., San Jose, CA, United States).

### 2.6 Histology

For histologic analysis, the lungs were isolated and fixed in formalin overnight. Afterward, the lungs were drained and embedded in paraffin at the pathology department at the university hospital in Erlangen. The samples were cut into 3 μm lung sections using a microtome (Leica, Wetzlar, Germany) and stained with H&E and PAS staining as well as collagen deposition by Sirius red and quantified (9, 34). The lung histologic sections, stained with H&E, were analyzed microscopically and evaluated in the pathology department by a pathologist and by the first author of this manuscript in a blinded manner according to the protocol of Doganci et al. (35). Therefore, a score of zero defines no detectable inflammation, a score of one is characterized by rare to occasional inflammatory cells around isolated peribronchial blood vessels, accumulations of scant inflammatory cells in more than one site is described as two, a score of three is defined as multifocal inflammation around peribronchial vessels, which are easily visible at ×4 magnification and four and as the most severe level, severe peribronchial inflammatory infiltrations at multiple sites (35). Lung sections stained with PAS and Sirius red were also analyzed microscopically and evaluated for mucus production in the bronchi of the mice’ lungs and collagen deposition was evaluate semi quantitatively around the bronchi. For histologic analysis of the gut, parts of the colon were fixed it in formalin overnight. It was drained and embedded in paraffin and cut into sections (3 μm). The inflammation score with H&E staining was based on the method of Erben et al. (36). Briefly, the density of leukocytes in the lamina propria was classified as 1 (<10%), 2 (10–25%), 3 (26–50%), and 4 (>50%). The extent of expansion of leukocytes infiltration was counted as 1 (mucosal), 2 (submucosal), 3 (transmural), and the sum of both values was used as inflammatory score (36).

### 2.7 Rhinovirus infection

After the murine model of HDM induced asthma, lung and splenic cells were used for infection with RV1b. Therefore, 500 μL RV suspension was applied to 10^6^ cells and incubated for 1 h at 33°C. After the incubation, cells were washed with 5 mL RPMI without supplements and up taken in cell culture medium. The cells were plated in a 48 well plate and cultured for 96 h. After harvesting the cells were used for RNA isolation and flow cytometry analysis. The mRNA expression of the virus was measured by qPCR with RV1b primers (5´-CCA TCG CTC ACT ATT CAG CAC-3′, 5´-TCT ATC CCG AAC ACA CTG TCC-3′) and normalized using the housekeeping gene hHPRT (5′-TGA CAC TGG CAA AAC AAT GCA-3′, 5′-GGT CCT TTT CAC CAG CAA GCT-3′).

### 2.8 ELISA

To analyze IL-5 levels in the serum mIL-5 ELISA set OptEIA (Cat. 555,236, BD Biosciences, Heidelberg, Germany) according to the manufacturer’s protocol was used. For measuring the total IgE antibody concentration in serum samples, an ELISA with antibodies against murine IgE (Cat. 555,248, BD Biosciences, Heidelberg, Germany) was performed according to the manufacturer’s protocol. Absorbance was measured with an ELISA Reader OPSYS MR (DYNEX Technologies, Denkendorf, Germany) at 450 nm.

### 2.9 Statistical analysis

**Figure 2:**
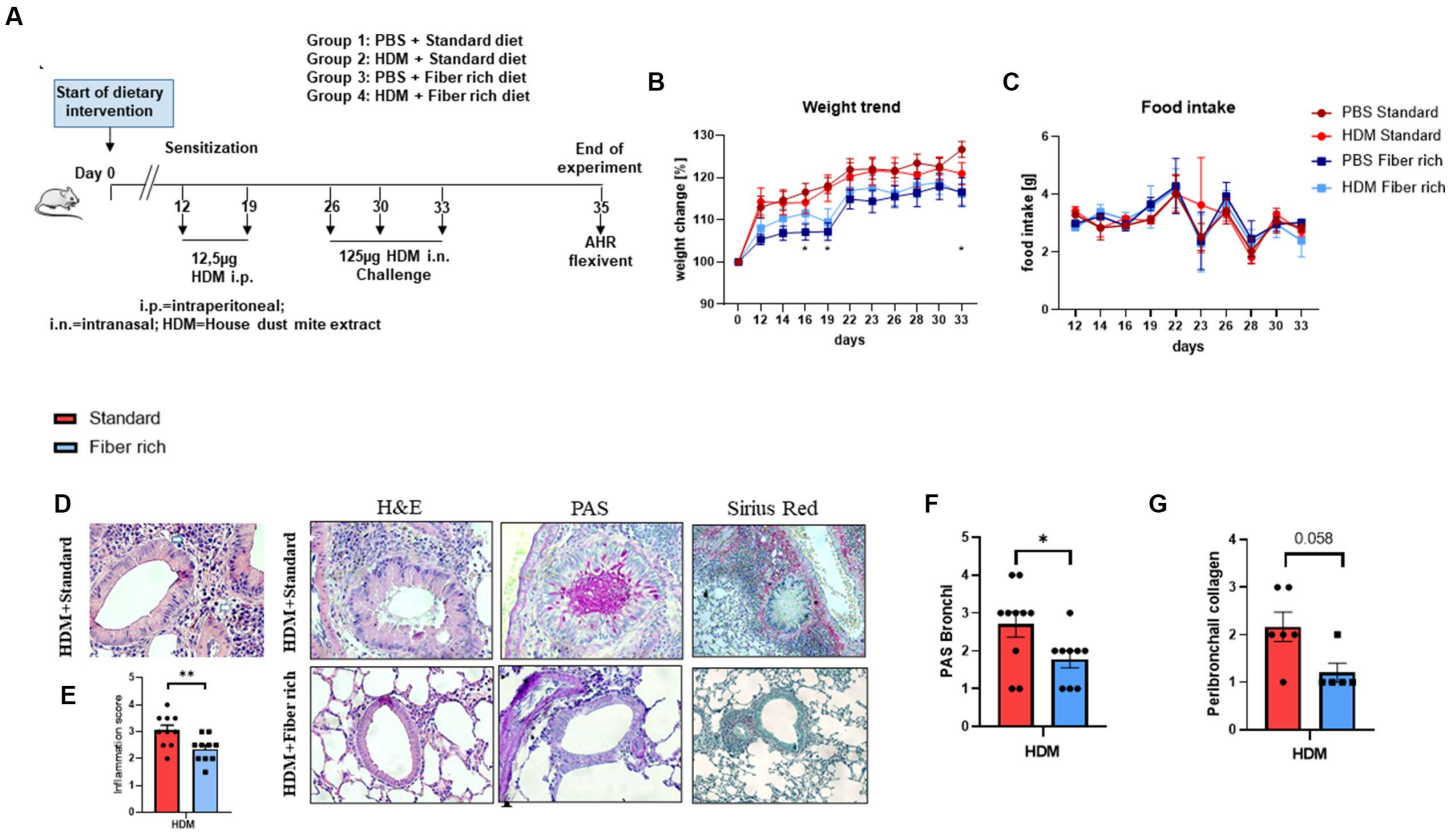
Asthmatic mice fed with fiber rich diet have significantly decreased lung inflammation, mucus production, and collagen deposition around the bronchi due to a high fiber diet. **(A)** Schematic presentation of the HDM-induced asthma model. Female 4-week-old Balb/c mice received different diets 1.5 weeks prior to the first HDM sensitization. On day 12 and 19, sensitization was performed by administrating intraperitoneally 12.5 μg HDM extract. On day 26, 30, and 33, the mice were challenged with 125 μg HDM intranasally. **(B)** Weight curves of all groups observed over the experimental time period as compared to the starting weight (100%) (PBS Standard vs. PBS Fiber rich: d16 *p* = 0.0371; d19 *p* = 0.011; d33 *p* = 0.0226; ^*^p < 0.05, *n* = 10). **(C)** Food intake of all groups monitored every second day by weighing the food of each cage (*n* = 10). **(D)** Representative histologic pictures taken in sections, stained with Hematoxylin and Eosin stain (left panels; 400×), PAS stain for mucus detection (middle panels; 400×), and with Sirius red-light-green (right panels; 200×). A representative picture for each experimental group is shown here. **(E)** Inflammation score according to Doganci et al. (35): “0: no inflammation; 1: rare occasional inflammatory cells around isolated peribronchial blood vessels; 2: accumulations of scant inflammatory cells around peribronchial vessels in more than one site; 3: multifocal inflammation around peribronchial vessels, easily visible at ×4 magnification; 4: severe peribronchial inflammatory infiltration at multiples sites” (*p* = 0.0096; ^**^p < 0.01, *n* = 10 animals per experimental group). **(F)** Lung tissue sections were stained with PAS and analyzed with a microscope at 400× magnification to detect mucus producing goblet cells. Semiquantitative analysis showed a significant reduction in mucus production by fiber rich food in asthmatic mice (*p* = 0.038, ^*^p < 0.05; *n* = 10, animals per experimental group). **(G)** Lung tissue sections were stained with Sirius red and fast green staining and analyzed with a microscope at 200× magnification to detect collagen deposition (in red) around the bronchi. Semiquantitative analysis showed a significant reduction in collagen deposition by fiber rich food in asthmatic mice (*p* = 0.058; *n* = 6 and 5). All data are shown as mean ± SEM.

**Figure 3:**
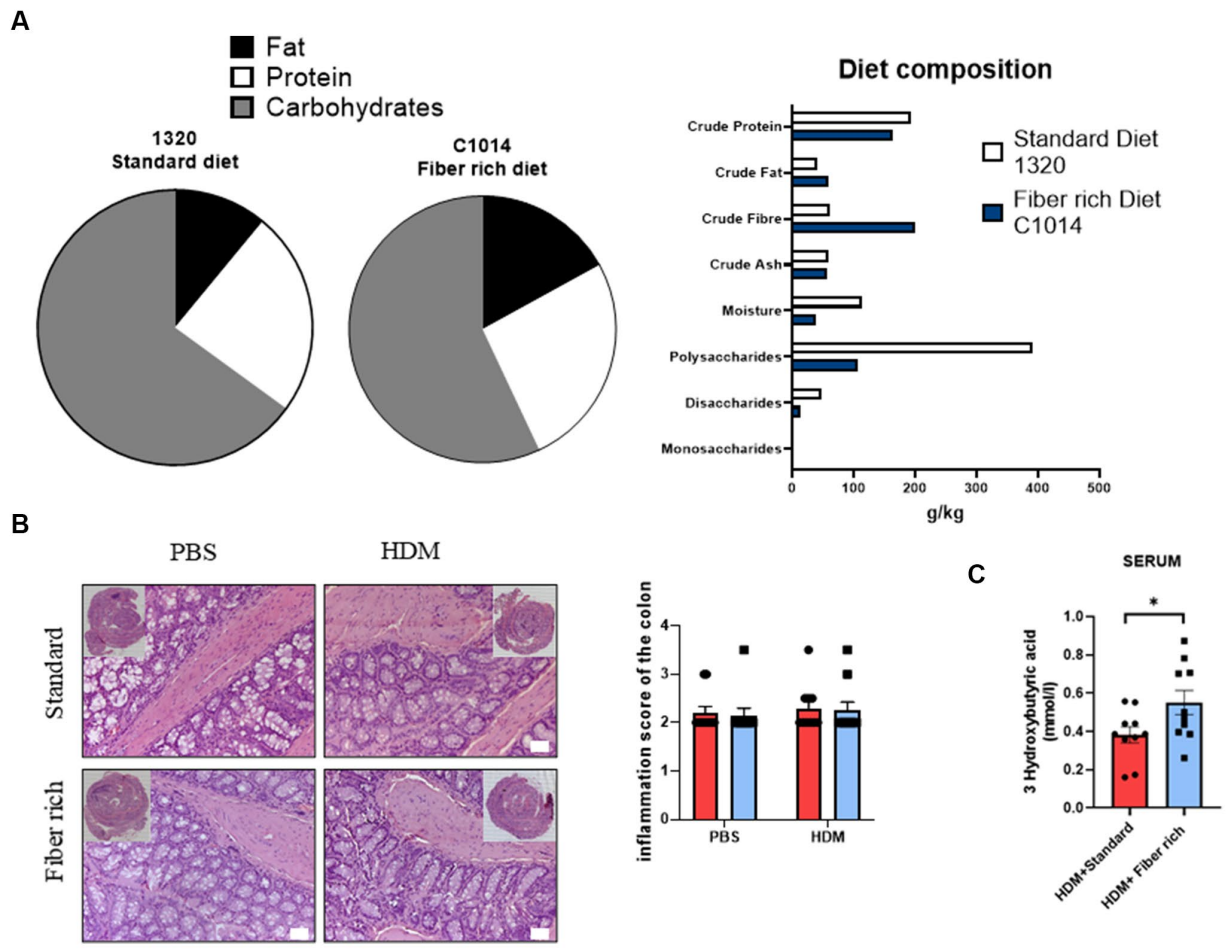
Asthmatic mice fed with fiber rich diet have induced SCFA (3-Hydroxybutyric acid) in serum as compared to asthmatic mice fed with standard diet. **(A)** Diet composition in accordance to the Altromin homepage informations. As standard control diet, the diet catalog number 1320 was used and for fiber rich diet C1004 was used. **(B)** Inflammation score of the colon analyzed according to Erben et al. (36). Representative pictures of Hematoxylin and Eosin stained gut sections are shown. **(C)** Measurements of 3-Hydroxybutyric acid in serum of asthmatic mice is shown (*p* = 0.040). Data are expressed as means ± SEM. The data were analyzed by unpaired *t*-test. ^*^p < 0.05, *n* = 10 animals per group.

**Figure 4:**
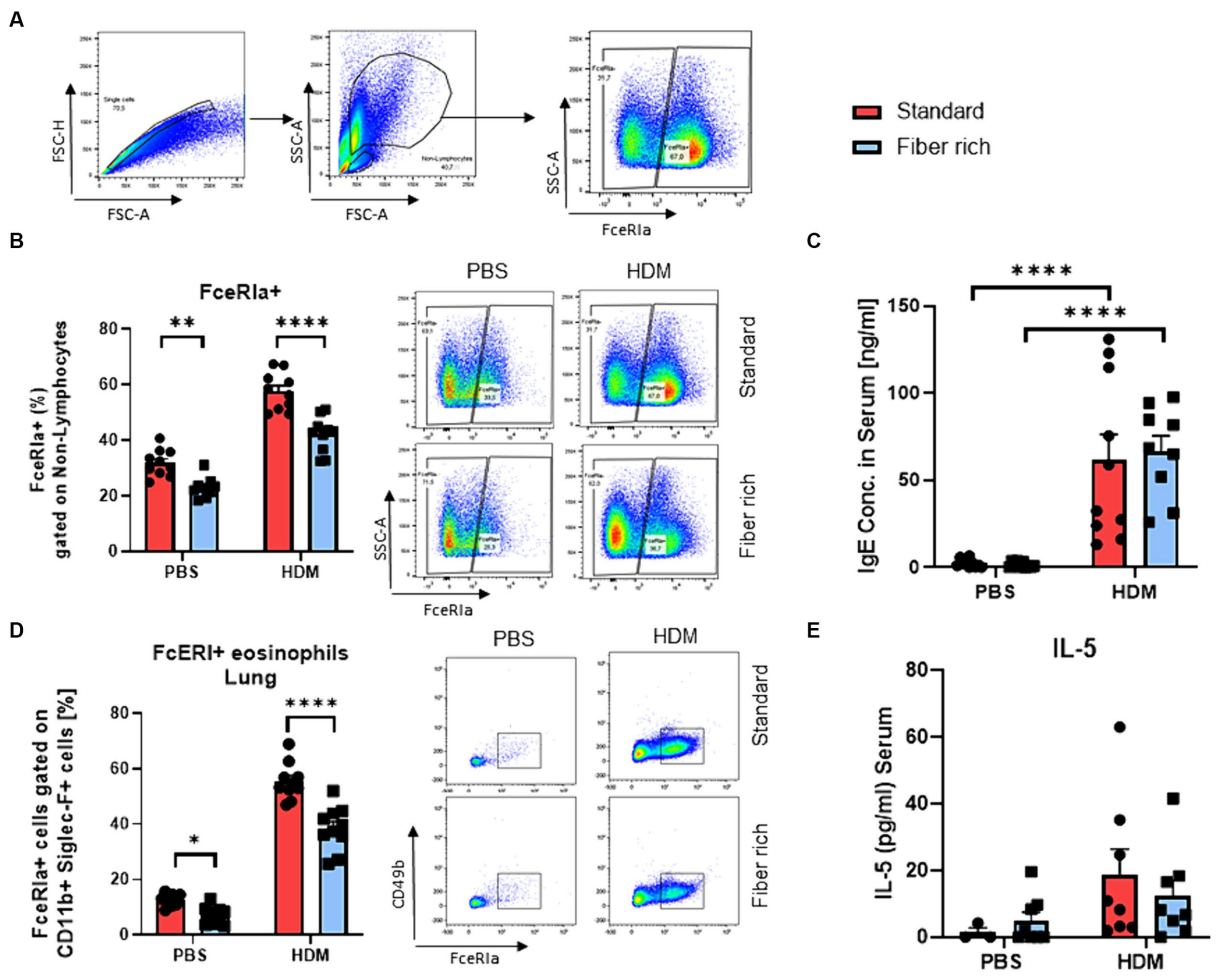
Fiber rich food significantly decreases the number of FcɛRIa + Eosinophils in the lungs of naïve and asthmatic mice. **(A)** Gating strategy for flow cytometry analysis of lung mast cells. **(B)** Analysis of FcɛRIa expression on non-lymphocytes via flow cytometry. (PBS Standard vs. PBS Fiber rich; *p* = 0.02, HDM Standard vs. HDM Fiber rich; *p* = 0.0001). A representative dot plot is shown. **(C)** ELISA analysis of total IgE in serum of PBS and HDM treated mice (PBS Standard vs. HDM Standard *p* < 0.001; PBS Fiber rich vs. HDM Fiber rich *p* < 0.001). **(D)** Analysis of lung FcɛRIa+ eosinophils along with representative dot plots (PBS Standard vs. PBS Fiber rich *p* = 0.0472; HDM Standard vs. HDM Fiber rich *p* < 0.0001). **(E)** Serum IL-5 Elisa. Data are expressed as means ± SEM. The data were analyzed by a two-way ANOVA and Sidak’s multiple comparisons test. ^*^p < 0.05, ^**^p < 0.01, ^****^p < 0.0001, *n* = 10 animals per group.

**Figure 5:**
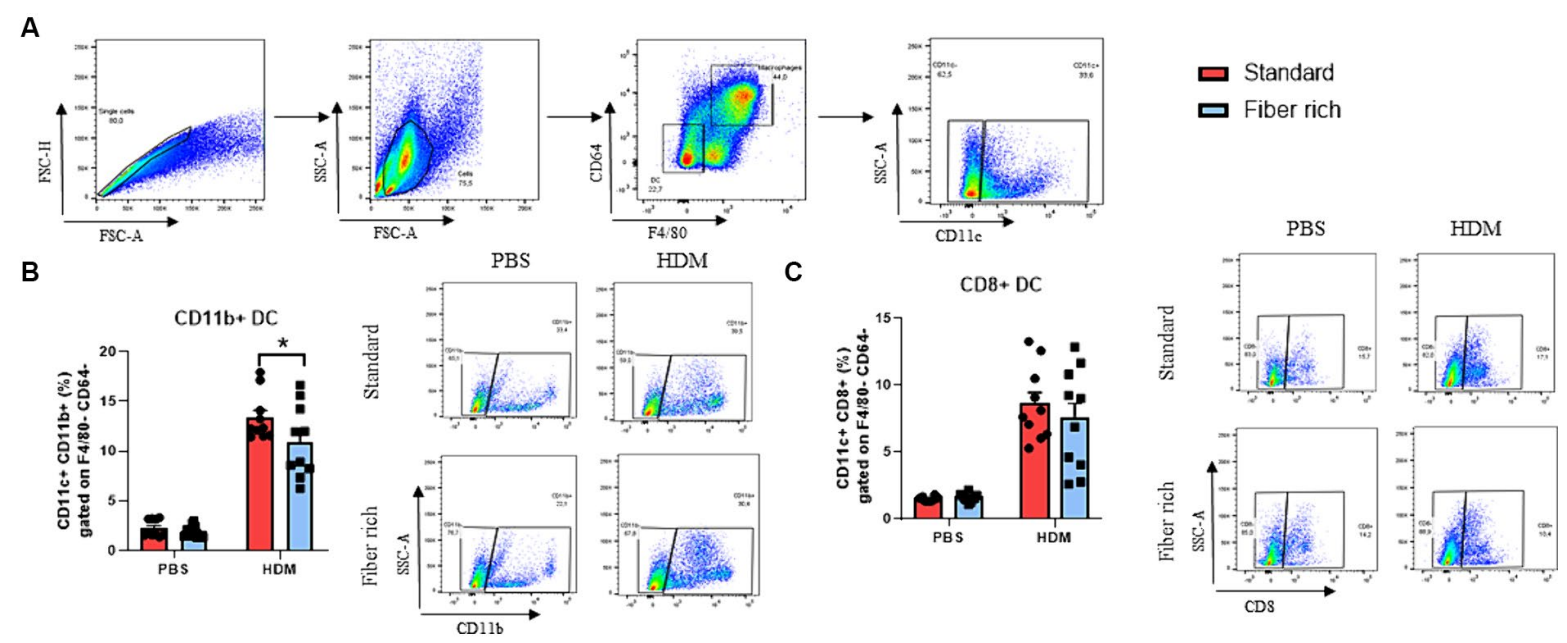
Fiber rich diet decreases the number of antigen presenting dendritic cells. **(A)** Gating strategy for flow cytometry analysis of lung dendritic cell subsets. **(B)** Percentages of CD11b + DC in the lungs of PBS and HDM animals (*p* = 0.0427). **(C)** Percentages of CD8+ DC in the lungs of PBS and HDM animals. Representative dot plots are shown. All data are presented as mean ± SEM. Representative dot plots are shown. The data was analyzed by two-way ANOVA, Sidak’s multiple comparisons test. ^*^p < 0.05, *n* = 10 animals per group.

**Figure 6:**
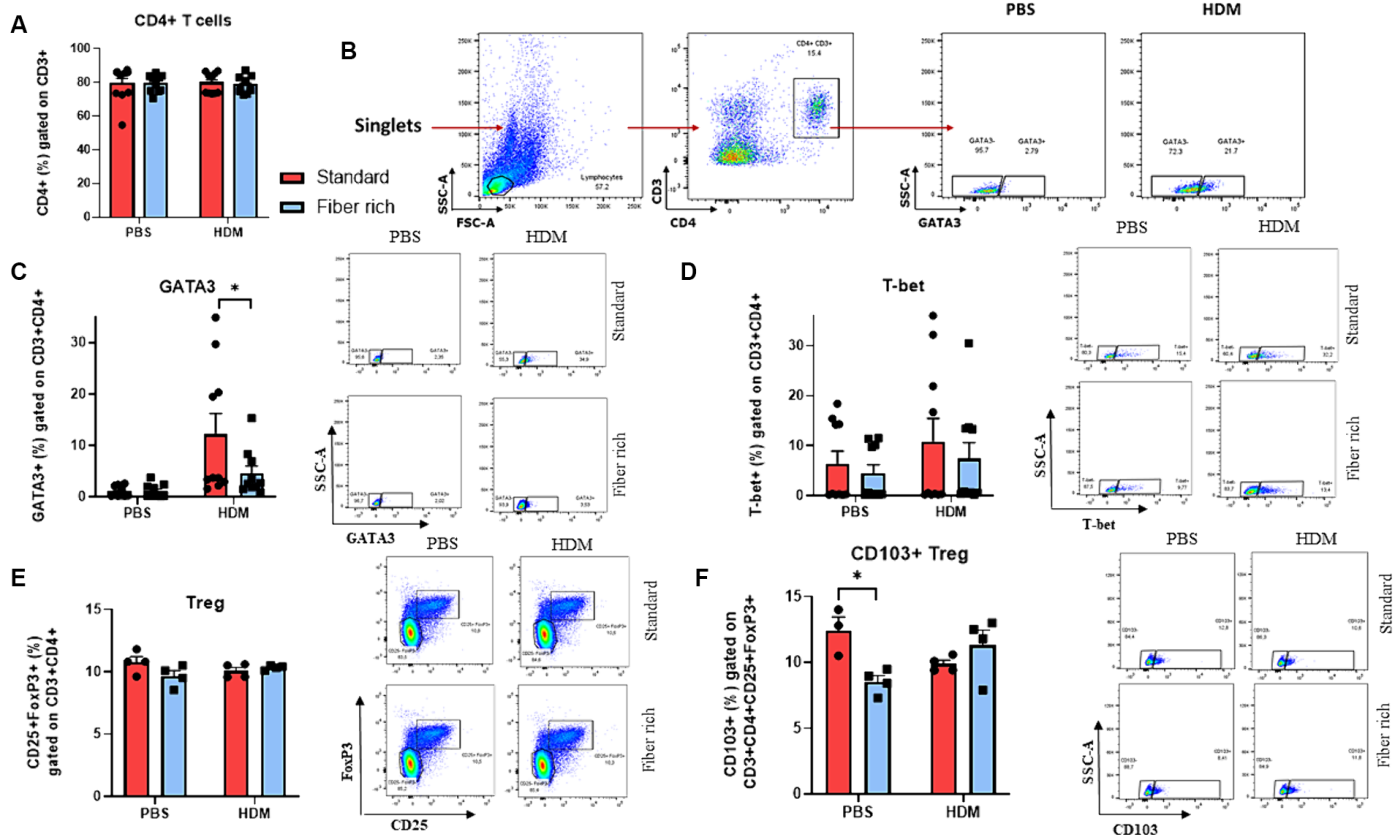
Suppression of GATA3+ CD4+ T cells in the lungs and changes in CD4 + CD25 + FoxP3 + CD103+ Tregs in the lymph nodes due to a fiber rich diet. **(A)** Analysis of CD4+ T cells in the lymph nodes. **(B)** Gating strategy of GATA3+ CD4+ CD3+ Th2 cells in the lung. **(C)** Flow cytometry analysis of GATA3+ CD4+ CD3+ Th2 cells in the lungs (*p* = 0.00338). **(D)** Flow cytometry analysis of T-bet+ CD4+ CD3+ Th1 cells in the lungs. **(E)** Flow cytometry analysis of CD4 + CD25 + FoxP3+ Tregs in the lungs of PBS and HDM mice. **(F)** Analysis of CD103+ Treg in the lymph nodes of asthmatic mice fed with high fiber diet or standard diet (*p* = 0.0139). All data are presented as mean ± SEM. Representative dot plots are shown. The data was analyzed by two-way ANOVA, Sidak’s multiple comparisons test. ^*^p < 0.05, *n* = 4–10 animals per group.

**Figure 7:**
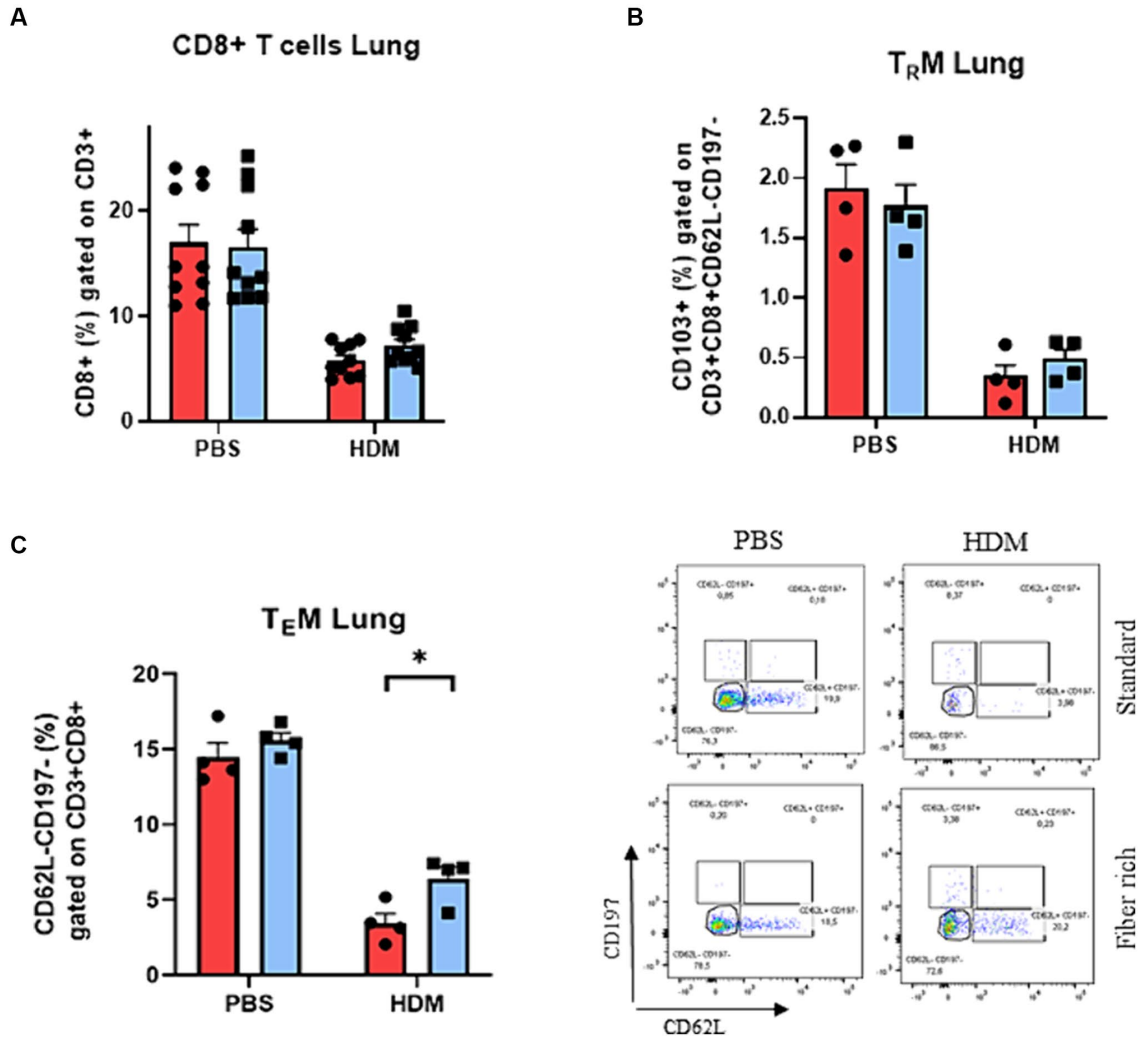
Increase of pulmonary TEMs of asthmatic mice fed with fiber rich diet. **(A)** Flow cytometry analysis of lung CD3 + CD8+ T cells. **(B)** Flow cytometry analysis of lung TRM characterized by CD103 expression (CD103 + CD62L-CD197-CD3 + CD8+). **(C)** Analysis of pulmonary TEMs (CD62L-CD197-CD3 + CD8+) via flow cytometry (*p* = 0.036). Representative dot plots are shown. All data are presented as mean ± SEM. Data are analyzed statistically by two-way ANOVA and Sidak’s multiple comparisons test. ^*^p < 0.05, *n* = 4–10 animals per group.

Statistical analysis was performed using the GraphPad Prism software V8 (GraphPad, La Jolla, CA, United States). All data are shown as mean with the standard error of the mean (SEM). Groups were statistically compared by either two-way ANOVA or unpaired *t*-test. The variables used in this study include continuous variables for Figures 1, 2B,C, 3A,C, 4–7. The variables in Figures 2E,F,G, 3B are categorical variables.

Figure 2E unpaired *t*-test, normal distribution, equal SD *p* = 0.0096;

Figure 2F Mann–Whitney U test *p* = 0.043;

Figure 2G Mann–Whitney U test *p* = 0.0584.

For *post-hoc*, a Sidak’s method test (two-way ANOVA) was used. Significant values are regarded at ^*^p < 0.05, ^**^p < 0.01, ^***^p < 0.001, and ^****^p < 0.0001.

## 3 Results

### 3.1 Reduced SCFA in the serum of children with asthma after disease exacerbation

The effects of the gut microbiota on asthma are partially mediated by bacteria metabolites, which may influence immune responses in distal parts of the body. The most known metabolites with demonstrated protective properties in human airway inflammation are SCFAs. Children with high amount of butyrate in the feces at 1 year of age are less likely to develop asthma at the age of 3 and 6 (37).

Thus this study asked if SCFA could be detected also in the serum of pre-school children. Therefore, β-Hydroxybutyric acid was measured in serum of the cohorts of pre-school children with and without asthma at recruitment visit into the Study Predicta (Figure 1A).

Here, considering that, airway symptomatic episodes of asthma could affect the SCFA formation by dysregulating gut microbiota and thus at baseline (B0) and convalescent visit, 6–8 weeks after symptomatic episodes, serum SCFA was measured and found that, the asthmatic children at convalescent visit exhibited a significant decrease of SCFA β-Hydroxybutyric acid in serum as compared to asthmatic and control children at the baseline visit (Figure 1B) (A-B0; *n* = 5) vs. A-C1, C2 (*n* = 11) *p* = 0.0218; C-B0 (*n* = 3) vs. A-C1, C2 (*n* = 11) *p* = 0.0109. In colors, paired data from the same child are shown. Further, the Predicta children were recruited 2 years later (F4) and asthmatic children were additionally analyzed after symptomatic visit at convalescent visit (A-C1, AC2) (Figure 1C). Also in this case we could confirm a decrease of β-Hydroxybutyric acid in serum of the asthmatic pre-school children at convalescence visit as compared to control children (Figure 1C). These data indicate, that exacerbation of disease in the airway could contribute to dysregulate the gut microbiota.

### 3.2 Asthmatic mice fed with a fiber rich diet have decreased asthma airway features

To further investigate the importance of a high fiber diet in asthma *in vivo*, a murine asthma model with HDM in combination with different diets with different fiber composition was set up. Therefore, 4-week-old Balb/c female mice received either fiber rich food modified with 30% pectin, or a standard diet as a negative control, one and a half weeks before the asthma model started (Figure 2A). To record weight changes and the food intake of all experimental groups, the mice and the food were weighed every second day during the experimental period. To calculate the weight change, the weight on the first day of the experiment was set up as 100%. Body weight was calculated with current weight/start weight. Thereby, lower body weights for mice that received the fiber rich diet compared to the mice that received standard diet was observed (Figure 2B) (PBS Standard vs. PBS Fiber rich: d16 *p* = 0.0371; d19 *p* = 0.011; d33 *p* = 0.0226).

Considering the food intake, a regression on day 23 and 26 was observed for all groups excluding at day 23 for the HDM treated mice fed with standard diet (Figure 2C).

To classify the severity of the HDM induced airway inflammation, lymphocytes and granulocytes infiltrations in lung tissue were analyzed microscopically at higher magnification. Therefore, sections of lung tissue derived from PBS and HDM treated mice which received either standard or fiber rich diet were stained with Hematoxylin and Eosin (H&E) to determine the severity of inflammation (Figure 2D, left hand side panels). The HDM treated mice fed with the standard diet showed an increase in infiltration of eosinophils as can be appreciated (Figures 2D,E, upper left panel). Furthermore, the HDM treated mice which received fiber rich food showed significantly less inflammation than the HDM treated mice fed with standard diet (Figures 2D,E, lower left panel) (*p* = 0.0096; ^**^p < 0.01, *n* = 10 animals per experimental group). Looking at the mucus production in the lungs of the mice by staining lung tissue sections with PAS (Figure 2D, middle panels), a visible and also significant decrease in mucus producing cells in the lungs of HDM treated mice which were fed with fiber rich food was detected (Figures 2D,F) (*p* = 0.038, ^*^p < 0.05; *n* = 10 animals per experimental group). Finally, collagen deposition by Sirius red staining analysis showed a decrease of collagen around the bronchi of fiber rich fed mice (Figure 2D, right panel and Figure 2G) (*p* = 0.058 *n* = 6 and 5).

### 3.3 Dietary fiber rich food induced serum concentration of 3-Hydroxybutyric acid in asthmatic mice

Next, the differences between the two types of diet were analyzed in more detail. Both diets contain crude fibers (Figure 3A). Fiber rich diet increases with 130% crude fiber in comparison to standard diet. Also, crude fat increases to 140% and crude protein appeared to be 15% less in fiber rich diet. The specific ingredients of the fiber rich diet (C1014-diet-Altromin) with 30% pectin can be found online. This diet includes the following raw materials: Sojamin, Corn starch, Cellulose, Sunflower oil, Premix of minerals and trace elements, Premix of vitamins, Pectin. Additionally, the inflammation score in colon sections stained with H&E did not change with diet changes (Figure 3B).

To examine the effects of the increased intake of dietary fiber, a closer look at serum concentration of 3-Hydroxybutyric acid in asthmatic mice was taken. 3-Hydroxybutyric acid, also named as β-Hydroxybutyric acid, is a metabolite that derives from butyric acid which is produced in the liver. Fiber rich diet caused a significant increase of the concentration of 3-Hydroxybutyric acid (Figure 3C) (*p* = 0.040).

### 3.4 Fiber rich food significantly decreased FcɛRIa+ eosinophils in the lungs of naïve and asthmatic mice

Moreover, a significant decrease in FcɛRI on non-lymphocytes in the PBS and asthmatic group fed with fiber rich food was found (Figures 4A,B). Second antigen contact leads to crosslinking of IgE bound on mast cells, resulting in mast cell degranulation, with release of histamine and other preformed bronchoconstrictors resulting in asthma exacerbations. Mast cells were defined as non-lymphocytes and further gated by CD49b, CD11b, c-kit (CD117), and FcɛRIa surface antibodies (Supplementary Table E2, Supplementary Figures S1A,B). No significant changes in mast cell numbers were observed between different diets in naïve and asthmatic mice. The IgE level in serum was found highly increased in HDM treated mice fed with standard as well as fiber rich diet compared to the control groups (Figure 4C). However, the number of eosinophils expressing FcɛRIa significantly decreased in the lungs of fiber rich fed mice (Figure 4D) plots (PBS Standard vs. PBS Fiber rich *p* = 0.0472; HDM Standard vs. HDM Fiber rich *p* < 0.0001). However, IL-5 did not change in the serum of fiber rich fed asthmatic mice (Figure 4E). In summary, the fiber rich food resulted in decreased FcɛRI in eosinophil expression. Further analysis on eosinophil function should be performed in future studies.

### 3.5 Fiber rich diet decreased the number of antigen presenting dendritic cells

Dendritic cells process antigens, present them to T effector cells and therefore regulate T cell activation including the activation of Th2 cells, which are key players in allergic immune responses. Here, classical dendritic cells of the lung stained with antibodies against F4/80, CD64, CD11c, CD8, and CD11b to differ between CD8+ and CD11b + DCs (Supplementary Table E2; Figure 5A) were analyzed. Here a significant decrease of CD11b + cDC2 in asthmatic mice fed with fiber rich food was found (Figure 5B). However, no differences in the numbers of CD8+ cDC1 could be detected (Figure 5C). These data indicate a reduced antigen presenting capability of lung DCs in asthmatic mice fed with fiber rich diet.

### 3.6 Fiber rich food significantly reduced the number of GATA3+ Th2 cells in HDM treated mice

Asthma shows a pathologic expansion of Th2 cells. To determine Th2 cells via flow cytometric analysis, cells were stained with antibodies against CD3, CD4, and GATA3 (Supplementary Table E2). It has also been reported that an imbalance of Th1/Th2 cells toward Th2 contributes to the pathogenesis of asthma (23–25). Here, although the CD4+ T cells did not change (Figure 6A), the number of GATA3+ CD3+ CD4+ cells was significantly decreased by fiber rich diet in asthmatic mice compared to HDM treated mice which received standard diet (Figures 6B,C) (*p* = 0.00338). Moreover, Th1 cells were analyzed as CD3, CD4, and Tbet+. However, there were no differences in Th1 cells due to fiber rich diet (Figure 6D). In conclusion, the anti-inflammatory effect observed in fiber rich diet is probably dependent on GATA3+ T cell downregulation in the airways of asthmatic mice.

### 3.7 Changes in CD4+CD25+Foxp3+CD103+ Treg in the lymph nodes of HDM treated mice due to a fiber rich diet

T regulatory cells (Tregs) are important for the immunosuppressive anti-inflammatory immune response. To define Tregs in flow cytometric analysis, total lung isolates and lymph nodes were gated as CD3+, CD4+, CD25+ and FoxP3+ cells (Supplementary Table E1, Figure 6E and Supplementary Figure S2). However, no difference in T Tregs were observed among the four groups. Furthermore, we analyzed CD103+ regulatory T cells in lymph nodes (Figure 6F) (*p* = 0.0139). This subset is supposed to suppress airway inflammation by producing high levels of immunosuppressive IL-10.

In lymph nodes a decrease in CD4+ CD103+ Foxp3+ T cells was detected in the PBS group due to fiber rich diet (Figure 6F).

### 3.8 T memory effector cells are induced by high fiber diet in asthmatic mice

Besides CD4+ effector T cells, also CD8+ effector T cells play an important role in the pathogenesis of asthma. To determine the subsets of CD8+ T cells, total lung cells were stained with antibodies against CD3, CD8, CD62L, CD197, and CD103 (Supplementary Table E2). CD103 is the determining marker for tissue resident memory CD8+ T cells (TRM) from effector memory CD8+ T cells (TEM). In general, a decrease in CD8+ T cell subsets in HDM induced asthma was observed (Figure 7A). Moreover, the number of pulmonary CD8+ T cells and TRMs did not change in asthmatic mice fed with fiber rich food (Figure 7B). By contrast, a significant induction of TEMs was detected in the lung of asthmatic mice fed with a fiber rich food compared to HDM treated mice which received standard diet (Figure 7C) (*p* = 0.036).

### 3.9 Fiber rich food diet controlled RV replication in the lung cells and induced CD8+ T cells in the spleen

**Figure 8:**
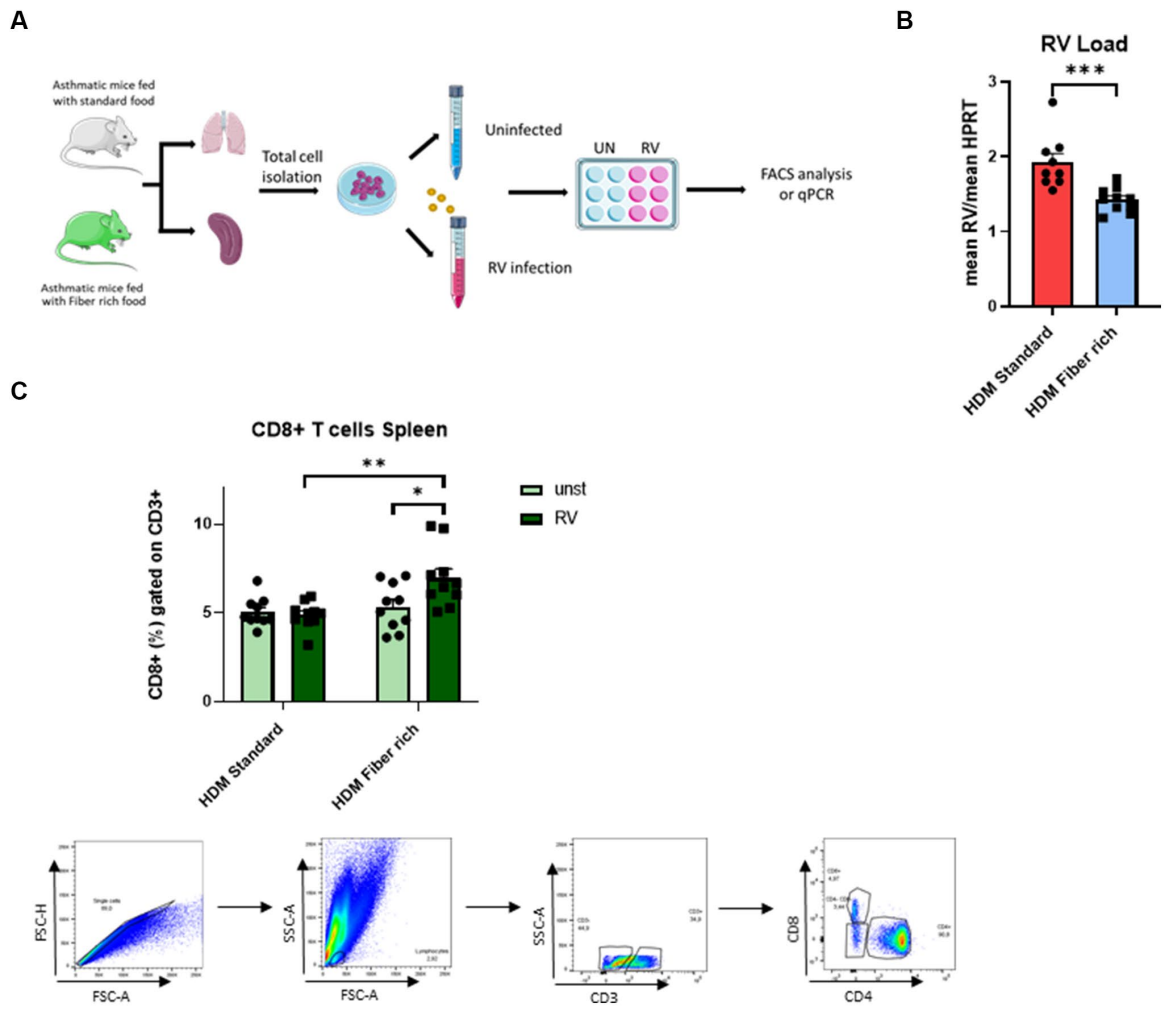
Induction of splenic CD8+ cells primed by fiber rich diet upon RV infection in asthmatic mice. **(A)** Experimental design for cell culture experiments. Lung and spleen cells from control or asthmatic mice fed with standard or fiber rich food were additionally infected with RV or remain uninfected and then incubated for 4 days at 37°C, 5% CO2. Afterward, the cells were harvested for flow cytometry analysis or RV mRNA analysis with qPCR. **(B)** RV mRNA in the infected lung cells was quantified by qPCR and normalized to reference gene HPRT (*p* = 0.0009). **(C)** Flow cytometry analysis of CD8+ T cells in total splenic cells infected with RV for 4 days or without infection (HDM Standard RV vs. HDM Fiber rich RV *p* = 0.0024; HDM Fiber rich unst. vs. HDM Fiber rich RV *p* = 0.0284) and gating strategy for flow cytometry analysis of spleen CD8+ T cells. The data were analyzed by two-way ANOVA, Sidak’s multiple comparisons test. ^*^p < 0.05; ^**^p < 0.01, ^***^p < 0.005; *n* = 7–10 per group.

In asthmatic children, symptomatic exacerbation of the disease are often associated with RV infection in their airways. Thus to mimic asthma exacerbation of disease, total lung cells from asthmatic mice fed with standard food or with fiber rich food were infected with RV (Figure 8A). Here, RV1b mRNA was significantly reduced in the lung cells from fiber rich food fed asthmatic mice as compared to those from standard food fed asthmatic mice (Figure 8B) (*p* = 0.0009). Looking for the underlying mechanism, an increase in CD8+ T cells in RV infected spleen cells from fiber rich fed mice was observed (Figure 8C) (HDM Standard RV vs. HDM Fiber rich RV *p* = 0.0024; HDM Fiber rich unst. vs. HDM Fiber rich RV *p* = 0.0284).

## 4 Discussion

It has been previously reported that dietary fibers may alter the gut microbiome and, thereby suppress systemic inflammation thus contributing to the resolution of asthma (21). Moreover, SCFAs which are produced by the gut microbiome by dietary fiber’s fermentation, can mediate anti-inflammatory effects of fiber rich food and hence ameliorate the pathogenesis of a range of diseases including asthma and allergic diseases (27). To investigate SCFA influence in asthma during exacerbations, SCFA was measured in the serum of pre-school children before and after asthma exacerbation at baseline and after symptomatic visit at convalescent visit as well as at the follow up visit. Here, SCFA concentration dropped in the serum of asthmatic children after disease exacerbations as compared to their baseline levels and to the level of control children. Interestingly, all the asthmatic children analyzed had a virus induced asthma. This indicated to us a possible influence of the respiratory infection on the gut microbiome composition during symptomatic episodes of asthma. Specifically, the gut microbiome regulated by respiratory viruses would reduce the fermentation of dietary fiber by inducing gut dysbiosis and thus downregulating the SCFA production in serum. The limitation of this observation is the small number of children analyzed. Thus, additional studies in enlarged cohorts should be undertaken with detailed microbiome and metabolome analysis. In experimental model of disease, lung inflammation was decreased in fiber rich fed asthmatic mice. This observation is consistent with the findings of Trompette et al. (21). Microscopically, a reduction of eosinophils, mucus and collagen production surrounding the bronchial wall was observed in asthmatic mice fed with fiber rich food.

Further investigations on lung function with mice fed a high fiber diet thus would be useful. In our model, dietary fiber reduced the body weight. This is consistent with the findings of Drew et al., where the intake of dietary fiber prevented obesity in mice with high fat diet (38). Also in humans, the consumption of dietary fiber is associated with reduced body weight and body fat (39). Accordingly, SCFAs were shown to stimulate the intestinal hormone peptide YY (PYY) by active action of FFAR3 which induces a feeling of satiety and therefore leads to reduced food intake (40). Moreover, fiber rich food induces changes in the gut microbiome resulting in the release of SCFA in the blood which influence inflammatory reaction going on in the lung after allergen exposure. Consistently, in asthmatic mice fed with fiber rich diet, a significant increase in serum 3-Hydroxybutyric acid was found. 3-Hydroxybutyric acid, which is also named as β-Hydroxybutyric acid, was found to be the major ketone body produced in the liver from fatty acids as a result of low carbohydrate/low calorie diet, which modulates the gut microbiota into “keto microbiota” (41). The pathways for ketone bodies are mainly ketogenesis and ketolysis and thus, take place in the mitochondrial matrix of hepatocytes. Free fatty acids are released from tissue under low insulin conditions and are then degraded to ketone bodies as ß-Hydroxybutyrate and acetoacetate after they are released into extrahepatic tissues for ketolysis. Ketone bodies also seem to show epigenetic modulation such as working as HDACs and exert anti-inflammatory effects providing potential for therapy in asthma (42). This finding is consistent with the increased concentration of 3-Hydroxybutyric acid since free fatty acids lead to the production of ketone bodies by ketogenesis (42). It has also been reported a direct correlation between β-Hydroxybutyrate and SCFAs. Therefore, 3-Hydroxybutyrate does not only take part in regulating cellular processes but also in altering the gut microbiome and inducing butyrogenesis. Sasaki et al. reported, that the consumption of β-Hydroxybutyrate leads to an increased production of SCFAs by gut microbiota (43). These data are consistent with our human data which might indicate that symptomatic exacerbation of the disease is accompanied with a long-lasting effect on SCFA production systemically that might affect the resolution of the disease.

To uncover the SCFA target cells in the lung, the FcεRIa expression which mediates the IgE cross-linking on mast cells, but is also present in basophils and other cells of innate responses was analyzed (44). Wang et al. described a decrease in degranulated mast cells and the release of inflammatory mediators in weaned pigs by administration of butyrate (45). In our model, a reduction of FcεRIa expression on eosinophils in the lungs of asthmatic mice fed with fiber rich food was detected. Nevertheless, when looking at the total IgE in serum of the mice, no reduction by dietary fiber fed asthmatic mice was observed. This stands in contrast to previous findings of Trompette et al. where IgE levels in serum are significantly reduced in mice fed with pectin rich diet (21). The differences could relate to the different models. In fact, Tropette’s studies were done in intranasal HDM challenge without intraperitoneal immunization. In conclusion, FcεRIa is significantly reduced by high fiber diet on eosinophils, whereas IgE remains unaltered. This might indicate decreased binding of IgE molecules to FcεRI receptor which is associated with less crosslinking and mast cell, basophil and eosinophil degranulation. In fact, crosslinking of FceRIa on eosinophils could lead to release of cytotoxic mediators responsible of the damage of the airway epithelial barrier seen in asthma. Thus, fiber rich food would also protect the airway epithelial barrier and thus reduce disease exacebations.Consistently with previous reports, a significant suppression of GATA3, the main Th2 transcription factor, expressing CD3+ CD4+ T cells in the lungs of asthmatic mice which were fed with dietary fiber was found (46, 47). It can therefore be assumed that Th2 cell response is suppressed by dietary fiber as a result of less antigen presentation of DCs leading to reduced airway inflammation. Cait et al. also showed effects of SCFAs produced by fermentation of dietary fiber modulating immune responses by dampening Th2 response in OVA treated mice which received vancomycin and SCFA treatment (46). Previous studies of Kespohl et al. reported a decrease of GATA3 expression in T cells via qPCR by treatment of 1 mM butyrate (47). It can be therefore assumed that Th2 cell response is suppressed by dietary fiber as a result of less antigen presentation of DCs and induction of Tregs leading to enhanced airway inflammation. In our study, significant suppression of cDC2 (CD11b + CD11c+) DC subset by dietary fiber was observed. This is consistent with the published anti-inflammatory effects of dietary fiber and the derived SCFAs. Dietary fiber may suppress airway inflammation by alteration of the gut microbiome and therefore inhibit the function of DCs by increasing circulating SCFA levels (22). It has been also reported that the administration of butyrate, decreases the ability of DC to prime Th2 response in dysbiotic mice (46). Accordingly, dietary fiber seems to suppress especially the Th2 priming cDC2 fraction thus, might inhibit their migration to lymph nodes and therefore suppress the ability to activate T lymphocytes toward the Th2 lineage. It has been reported that a lack of Tregs or Treg dysfunction might not effectively suppress Th2 immune response, allergic diseases and thus asthma can develop (48). Tregs expressing the integrin CD103 have been identified as high IL-10 producing Tregs which also seem to demonstrate enhanced suppression for Th2 mediated allergic responses (49). In our studies, no changes in Tregs in asthmatic mice due to a fiber rich diet were detected.

Antigen presenting cells are required to prime CD8+ T cells for involvement in immune responses against pathogens and viral infections. Naïve CD8+ T cells are therefore activated and express T cell receptors. In our study, dietary fibers affected CD8+ effector cells and thus, influenced the capacity of CD8+ effector T cells to differentiate into CD8+ memory cells. TRMs unlike TEMs are located in specific tissues, especially mucus organs as the lungs or intestine, whereas TEMs recirculate through non-lymphoid tissues. TRMs promote the secretion of the pro-inflammatory cytokine IL-2. However, they are also able to produce IL-10, which leads to dampened inflammation and the resulting tissue damage. TEMs are at first case able to fight against infections which develop in peripheral tissues because of their ability to localize in those tissues and also due to their cytotoxicity (50). Nevertheless, Marsland et al. reported that CD8+ memory T cells may play an important role not only during viral infections, but also in allergic airway inflammation. CD8+ memory T cells therefore seem to inhibit allergen-induced airway inflammation by producing TGF-ß which acts suppressive against immune responses, reduce activation level, AHR and Th2 cytokine production (51). Previous studies, which investigated the dietary regulation on memory T cells, also showed that the microbiota altered by dietary fiber is also able to influence the development of memory CD8+ T cells. This was demonstrated by mice which received a 35% crude fiber diet. Mice with the high intake of dietary fiber showed an increased development of CD8+ memory T cells during a herpes-simplex virus infection (52). A consistent increase in TEMs in the lungs in asthmatic mice which was mediated by fiber rich diet was found. Finally, a decreased RV replication induced in the lung cells from fiber rich fed mice was detected. Further, this anti-viral effect in the lungs was associated to induction of cytotoxic CD8+ T cells. Regarding this, Trompette et al. reported a promotion of differentiation and activation of CD8+ T cell response by dietary fiber as well as derived SCFAs during influenza virus infection by activation of FFAR3 and altered metabolism in fatty acid oxidation (53). So dietary fiber or as well as derived SCFAs seem to increase antiviral immunity through CD8+ T cell activation. The ability of high fiber intake to modulate immune defense against respiratory viral infections also displays the measured virus load. Further, a significant decrease of virus load in lung after 4 days of infection which was mediated by a fiber rich diet was detected in this study. This is also consistent with the findings of Trompette et al., who described a decreased viral load in mice fed with high fiber diet after 8 and 10 days of influenza infection (53). This further indicates the beneficial effects of dietary fiber derived SCFAs which modulate immune response during allergic and also virus induced airway inflammation.

In summary, SCFA production might play an important role during virus mediated exacerbation of asthma in preschool children as this pathway is suppressed by the respiratory infection. This is a novel finding that needs to be followed up in enlarged cohorts. Consistently, fiber rich diet had resolving effects on airway inflammation, mucus production and collagen deposition and local Th2 response during experimental asthma development by reducing antigen presentation of cDC2, the GATA3 Th2 effector cells. Moreover, a significant downregulation of lung eosinophils carrying the FcɛRIa was observed. Additionally, CD8+ effector memory T cells were boosted by fiber rich diet. This was also confirmed in a model of RV infection *ex vivo*. Although this study should be expanded to more specific cellular analysis, it demonstrates a need for clinical investigations on the effects of fiber rich diet on the asthmatic burden of human patients as a possible supportive therapy option especially during asthma exacerbations.

## Data availability statement

The original contributions presented in the study are included in the article/Supplementary material, further inquiries can be directed to the corresponding author.

## Ethics statement

The European study PreDicta (Post-infectious immune reprogramming and its association with persistence and chronicity of respiratory allergic diseases) assessed two cohorts of healthy and asthmatic preschool children aged 4–6 years old. The study was approved by the ethics committee of the Friedrich-Alexander University (FAU) Erlangen-Nürnberg, Germany (Re-No 4435) and is registered in the German Clinical Trials Register (www.germanctr.de: DRKS00004914). The studies were conducted in accordance with the local legislation and institutional requirements. Written informed consent for participation in this study was provided by the participants’ legal guardians/next of kin. The experiments were approved by the local animal ethics committee Regierung von Unterfranken (AZ: 55.2.2-2532-2-633). The study was conducted in accordance with the local legislation and institutional requirements.

## Author contributions

AS: Conceptualization, Data curation, Formal analysis, Investigation, Methodology, Software, Visualization, Writing – original draft, Writing – review & editing. AG: Methodology, Writing – review & editing. EN: Methodology, Writing – review & editing. ZY: Formal analysis, Investigation, Methodology, Software, Writing – review & editing. SK: Formal analysis, Investigation, Methodology, Software, Visualization, Writing – review & editing. AL: Methodology, Writing – review & editing. A-KB: Methodology, Writing – review & editing. ST: Methodology, Writing – review & editing. SM: Methodology, Writing – review & editing. MR: Methodology, Writing – review & editing. CG: Methodology, Writing – review & editing. PT: Methodology, Writing – review & editing. KH: Methodology, Writing – review & editing. RR: Methodology, Writing – review & editing. OS: Methodology, Writing – review & editing. SZ: Formal analysis, Methodology, Writing – review & editing. SF: Conceptualization, Data curation, Formal analysis, Funding acquisition, Investigation, Methodology, Project administration, Resources, Software, Supervision, Validation, Visualization, Writing – original draft, Writing – review & editing.
