# Supplementary material for: Fiber rich food suppressed airway inflammation, GATA3 + Th2 cells, and FcεRIα+ eosinophils in asthma
Source: Front Nutr. 2024 May 2;11:1367864. doi: 10.3389/fnut.2024.1367864 (PMC11097976; doi:10.3389/fnut.2024.1367864)

Supplementary Material

Fiber rich food suppressed airway inflammation, GATA3+Th2 cells and FcεRIα+ eosinophils in asthma

Alicia Schenzel[1*], Adriana Geiger[1], Elvedina Nendel[1], Zuqin Yang[1], Susanne Krammer[1**], Anna Leberle[1], Ann-Kathrin Brunst[1], Sonja Trump[1], Susanne Mittler[1], Manfred Rauh[2], Carol I. Geppert[3,4,5], Patrick Tausche[1***], Katja Hohenberger[1***], Ralf J. Rieker[3,4,5], Oliver Schieweck[6], Sebastian Zundler[4,7], and Susetta Finotto[1,4,5,8*&].**

[1]Department of Molecular Pneumology, Friedrich-Alexander-University (FAU) Erlangen-Nürnberg, Universitätsklinikum Erlangen, 91052 Erlangen, Germany

[2]Children's Hospital, Department of Allergy and Pneumology, Friedrich-Alexander-University (FAU) Erlangen-Nürnberg, Universitätsklinikum Erlangen, 91054 Erlangen, Germany

[3] Institute of Pathology, Friedrich-Alexander-University (FAU) Erlangen-Nürnberg, Universitätsklinikum Erlangen, 91052 Erlangen, Germany

[4] Deutsches Zentrum für Immunotherapie (DZI)

[5]Bavarian Cancer Research Center (BZKF), Erlangen.

[6]Laboratory of Clinic Medicine, Friedrich-Alexander-University (FAU) Erlangen-Nürnberg, 91052 Erlangen, Germany

[7] Department of Internal Medicine 1, Friedrich-Alexander-University (FAU) Erlangen-Nürnberg, Universitätsklinikum Erlangen, 91054 Erlangen, Germany

[8]Comprehensive Cancer Center Erlangen-EMN (CCC ER-EMN), Erlangen, Germany

***contributed equally to this work and thus share the first authorship**

****,*** contributed equally to this work**
[&] Correspondence:
Prof. Dr. Dr. Susetta Finotto

Universitätsklinikum Erlangen

Abt. Molekulare Pneumologie

Hartmannstraße 14

91052 Erlangen

Phone: +49-9131-85-35883

Mail: susetta.finotto@uk-erlangen.de

http://www.molekulare-pneumologie.uk-erlangen.de

1 Supplementary Data

1.1 Airway hyperresponsiveness

On day 35 invasive measurement of AHR was performed. After deeply euthanizing the mice via intraperitoneal injection with pentobarbital, tracheotomy was performed and a cannula (18G) was inserted into the trachea. The mice were then connected with the flexivent system (EMKA, Paris, France) and AHR was measured by exposing the mice to increasing doses of Methacholine (MKCK9552, Sigma-Aldrich, St. Louis, USA) (0mg/ml (PBS); 12.5mg/ml; 25mg/ml; 50 mg/ml).

1.2 Serum preparation from blood

Before organs were removed, blood was collected using a syringe. The blood was transferred to a 1.5ml Eppendorf tube and centrifuged (600xg RT 30min) to receive the serum phase. Serum was removed and transferred to another tube and immediately stored at -80°C.

1.3 Total cell isolation of lung

Total lung cell isolation was performed after overnight storage in MACS Tissue storage solution (Miltenyi Biotec, Bergisch Gladbach, Germany). Therefore, the lungs were transferred to a petri dish and minced into small pieces. For digestion of the lung, the organ was incubated in 10ml of Collagenase/DNAse at 37°C for 45 min (Cat: C98991, Sigma-Aldrich, St. Louis, USA; Cat: 10104159001, Roche Diagnostics, Basel, Suisse). Afterwards the digested lungs were push through a cell strainer (40µm) into a 50ml falcon to obtain a single cell suspension. The cell strainer was rinsed with 5ml RPMI 1640 medium (without supplementation) (AC-LM-0060, Anprotec, Bruckberg, Germany) to wash remaining cells. Next, the isolated cell suspension was centrifuged (1500rpm 4°C 10 min) and the pellet was resuspended in 10ml ACK-Lysis (8.29g NH_4Cl, 1g $KHCO_3$ and 0.367g Na_2 – EDTA dissolved in 1L distilled water and at the end the pH value was adjusted with NaOH to 7.2 – 7.4) for two minutes at room temperature (RT) to lyse red blood cells. After another centrifugation (1500rpm 4°C 5 min), the cell pellet is resuspended in 10ml PBS (RPMI supplemented with 10% FCS, 5% PenStrep, 5% L-Glutamine) and fat was removed by slowly pipetting the suspension. After centrifugation (1500rpm 4°C 5 min), the supernatant was discarded, and the cell pellet resuspended in 5ml medium to count the cells subsequently. Cell counting was performed by using a Neubauer counting chamber. Lymph nodes single cell suspension was isolated immediately after isolating the lymph nodes. Therefore, the lymph nodes were also pushed through a cell strainer (40µm) into a 50ml falcon to obtain a single cell suspension and washed with 5ml RPMI Medium. After centrifugation (1500rpm 4°C 5 min), the cells were taken up into 5ml RPMI medium again and counted in the same manner as the lung cell suspension.

Supplementary Tables

Table E1:Serum B-hydroxybutiric acid (mmol/L) analysis in the cohorts of children with asthma (A) and control children without asthma (C).

C-B0	A-B0	C-F4	A-F4	A-C1	A-C2
0,02	0,03	0,01	0,05	0,03	0,04
0,14	0,03	0,07	0,02	0,03	0,03
0,08	0,09	0,03	0,04	0,03	
	0,04	0,04	0,05	0,03	
	0,13	0,04	0,02	0,02	
		0,03	0,02	0,02	
		0,02	0,03	0,02	
		0,03	0,05	0,05	
		0,18		0,04	
		0,03		0,02	
		0,04			
		0,02			
		0,04			

B0= baseline visit; F4= follow up visit at 24 months;

C1 and C2 = convalescent visit 6-8 weeks after disease exacerbations (symptomatic visit).

Table E2. FACS antibodies used in this study

Antigen	Species	Fluorochrome	Dilution	Catalog number	RRID	Manufacturer
Fc-Block (anti-CD16/CD32)	Mouse	Unlabeled	1:100	553142	AB_394657	BD Biosciences
CD3e	Mouse	PE-Cy7	1:200	552774	AB_394460	BD Biosciences

CD3e	Mouse	V450	1:150	560804	AB_2034004	BD Biosciences
CD4	Mouse	APC	1:100	130-109-415	AB_2657960	Miltenyi Biotec
CD4	Mouse	BV421	1:200	740007	AB_2739779	BD Biosciences
CD8a	Mouse	PerCP	1:150	100732	AB_893423	BioLegend
CD8a	Mouse	BV510	1:150	563068	AB_2687548	BD Biosciences
CD8a	Mouse	PE-Vio770	1:150	130-109-249	AB_2659500	Miltenyi Biotec
T- bet	mouse/human	BV421	1:50	563318	AB_2687543	BD Biosciences
GATA3	Mouse	PE	1:50	560074	AB_1645330	BD Biosciences
CD25	mouse	APC – Fire 750	1:200	557658	AB_396773	BD Biosciences
CD62L	Mouse	PE-Cy5	1:200	15-0621-81	AB_468766	invitrogen
CD197 (CCR7)	Mouse	PE	1:100	560682	AB_1727442	BD Biosciences
CD103	mouse	FITC	1:150	130-102-479	AB_2654397	Miltenyi Biotec
FoxP3	Mouse	AlexaFluor 647	1:50	560402	AB_1645202	BD Biosciences
CD11c	Mouse	APC-Cy7	1:200	117324	AB_830649	BioLegend
CD11b	Mouse	V450	1:200	560456	AB_1645267	BD Biosciences

CD11b	Mouse	APC-Vio770	1:100	130-109-366	AB_2654656	Miltenyi Biotec
F4/80 (EMR1)	Mouse	PerCP	1:200	123126	AB_893483	BioLegend
CD64	Mouse	PE-Cy7	1:200	139313	AB_2563903	BioLegend
CD117 (c-kit)	Mouse	APC	1:200	17-1171-82	AB_469430	eBioscience
CD49b	Mouse	FITC	1:200	553857	AB_395093	BD Biosciences
FceRIa	Mouse	PE-Cy7	1:200	134317	AB_10643996	BioLegend
Siglec-F	Mouse	BV510	1:150	740158	AB_2739911	BD Biosciences
Gr-1 (Ly-6G)	Mouse	PE	1:200	130-102-426	AB_2659861	Miltenyi Biotec
CD45	Mouse	APC-Cy7	1:150	557659	AB_396774	BD Biosciences

2 Supplementary Figures

Figure S1: AHR was not altered by fiber rich diet (A) Invasive lung function measurement with increasing doses of methacholine. All data is shown as mean ± SEM from 6-10 animals per group. Statistical analysis was performed using a two-way ANOVA and Sidak's multiple comparisons test. (B) Gating strategy of Flow cytometry analysis for lung granulocytes. A representative dot plot is shown. (C) Flow cytometry analysis of lung cells. A representative dot plot is shown. All data is presented as mean ± SEM. Data is analyzed statistically by two-way ANOVA and Sidak's multiple comparisons test. n=6-10 animals per group.

Figure S2: Regulatory CD4+CD25+Foxp3+ T cells in the lungs by fiber rich food in naïve and HDM treated mice. (A) Gating strategy for lung staining with antibodies against CD3, CD4, CD25 and FoxP3 to distinguish Tregs. (B) Analysis of CD4+ T cells in the lungs. (C) Flow cytometry analysis of Tregs in the lungs of control and asthmatic mice fed with fiber rich diet or standard diet. Representative dot plots are shown. All data is presented as mean ± SEM. Data is analyzed statistically by two-way ANOVA and Sidak's multiple comparisons test. n=4-10 animals per group.

Supplementary Figure S1

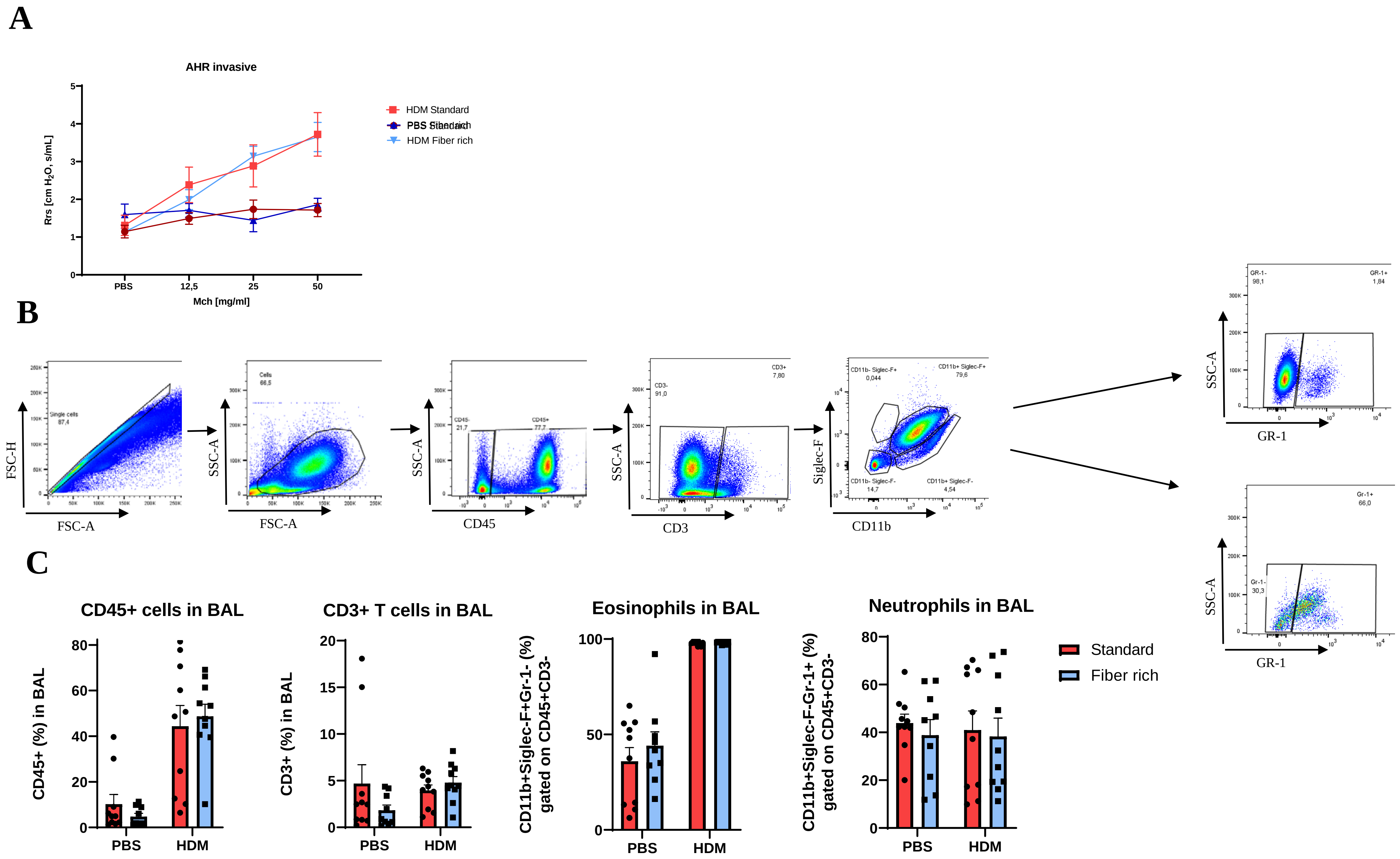

Supplementary Figure S2

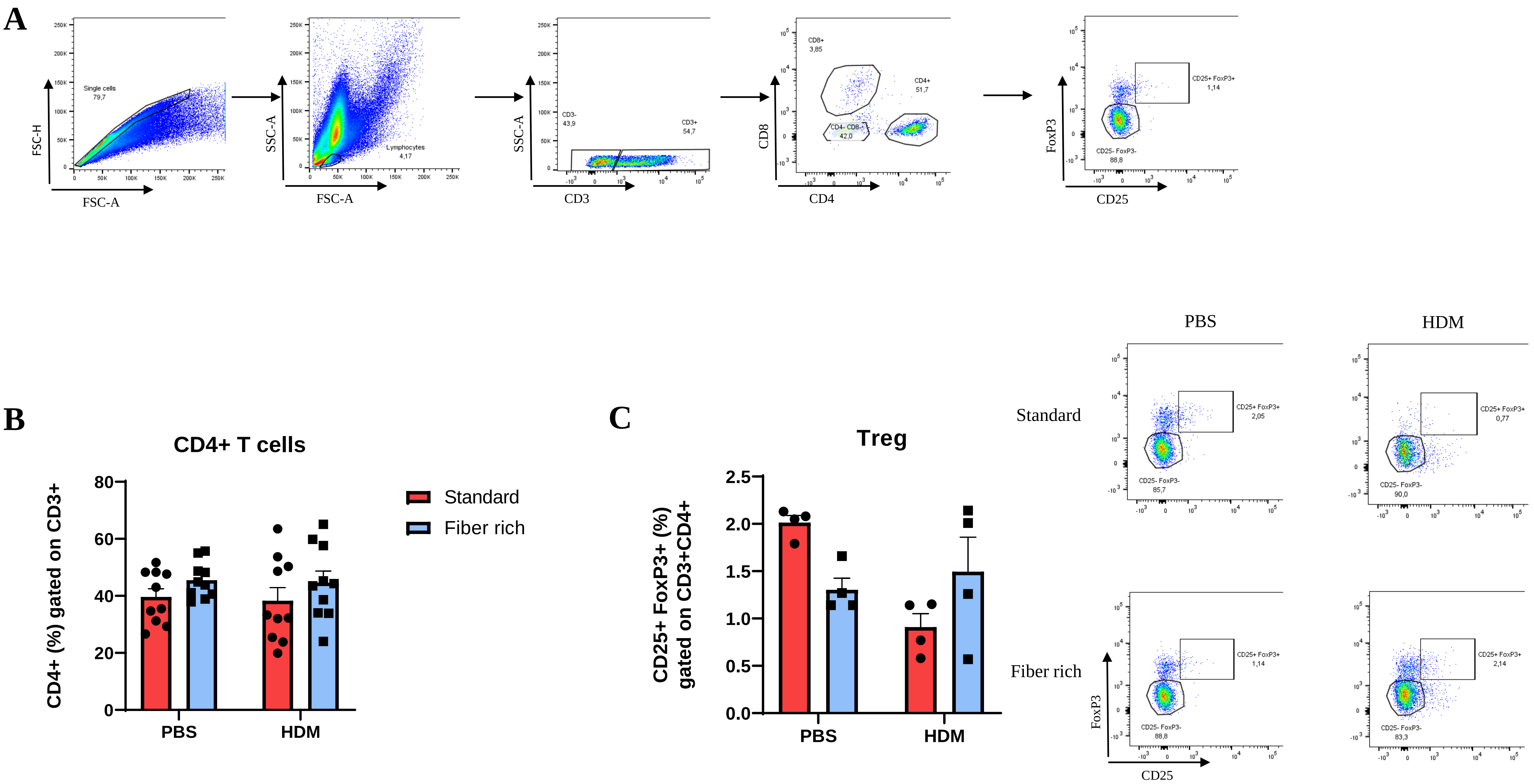

Supplement: Supplementary file 1 [file Data_Sheet_1.pdf]
